# Substrate Adhesion Regulates Sealing Zone Architecture and Dynamics in Cultured Osteoclasts

**DOI:** 10.1371/journal.pone.0028583

**Published:** 2011-12-05

**Authors:** Fabian Anderegg, Dafna Geblinger, Peter Horvath, Mirren Charnley, Marcus Textor, Lia Addadi, Benjamin Geiger

**Affiliations:** 1 Laboratory for Surface Science and Technology, Department of Materials, ETH Zurich, Zurich, Switzerland; 2 Department of Structural Biology, Weizmann Institute of Science, Rehovot, Israel; 3 Department of Molecular Cell Biology, Weizmann Institute of Science, Rehovot, Israel; 4 Department of Biology, Light Microscopy Center, ETH Zurich, Zurich, Switzerland; University of Birmingham, United Kingdom

## Abstract

The bone-degrading activity of osteoclasts depends on the formation of a cytoskeletal-adhesive super-structure known as the sealing zone (SZ). The SZ is a dynamic structure, consisting of a condensed array of podosomes, the elementary adhesion-mediating structures of osteoclasts, interconnected by F-actin filaments. The molecular composition and structure of the SZ were extensively investigated, yet despite its major importance for bone formation and remodelling, the mechanisms underlying its assembly and dynamics are still poorly understood. Here we determine the relations between matrix adhesiveness and the formation, stability and expansion of the SZ. By growing differentiated osteoclasts on micro-patterned glass substrates, where adhesive areas are separated by non-adhesive PLL-*g*-PEG barriers, we show that SZ growth and fusion strictly depend on the continuity of substrate adhesiveness, at the micrometer scale. We present a possible model for the role of mechanical forces in SZ formation and reorganization, inspired by the current data.

## Introduction

The ability of adherent cells to sense the chemical and physical properties of the extracellular matrix (ECM), and the integration of this information, play critical roles in regulating cell morphology, migration, division, differentiation and survival [1], [2], [3], [4], [5], [6]. To explore the effects of specific chemical and physical substrate features on the adhering cells, micro- and nanostructured surfaces with defined chemical and physical properties were employed as adhesive cell matrices [7], [8]. In such experimental systems, it was possible to vary individual surface features such as the chemical composition of the surface [7], its dimensionality [9], [10], topography [11], [12], ligand density [13], [14] and rigidity [2], [6], and measure the resulting cellular responses. In this study, we used micro-patterned substrates to address the role of cell adhesion in the local and global regulation of the formation and dynamics of the resorptive apparatus of cultured osteoclasts.

In vertebrates, bone undergoes continuous remodelling cycles, whereby osteoblasts and osteoclasts regulate bone formation and degradation in a coordinated manner. Osteoblasts are primarily responsible for the *de novo* synthesis of bone, whereas the monocyte-derived, multi-nucleated osteoclasts are responsible for bone degradation. The two processes must be tightly balanced in order to guarantee proper bone homeostasis and calcium metabolism [15], and loss of this balance leads to severe pathological conditions, such as osteoporosis [16] and other skeletal disorders [17].

The bone-resorbing function of osteoclasts is dependent on the formation of an actin-rich sealing zone (SZ), through which osteoclasts tightly adhere to the bone surface [18]. SZs constitute diffusion barriers, delimiting the ventral ruffled border, which is primarily responsible for bone resorption through secretion of protons and proteolytic enzymes into the underlying resorption lacuna, and the removal of the degradation products [19], [20].

The SZ constitutes a highly interconnected ring-like structure formed by podosomes, the adhesive building blocks in osteoclasts [21], [22]. Individual podosome architecture consists of a central core bundle of F-actin filaments, surrounded by a ring of integrins, and associated adhesion plaque proteins, such as vinculin and paxillin [21]. Upon SZ formation, the constituent podosomes become tightly cross-linked by interconnecting actin filaments, and their adhesive domains reorganize into an inner and an outer ring of plaque proteins, supporting the view that these rings serve as major adhesive structures in differentiated osteoclasts [23], [24], [25], [26]. The entire process of osteoclast maturation can be visualized in cultured osteoclasts, consisting in the assembly of individual podosomes into dynamic clusters that further evolve into small rings, which eventually fuse to form a large, stable SZ-like structure [27], [28]. Live-cell imaging shows that these SZs expand centrifugally by forming new podosomes at their outer periphery, while dissociating those located along the inner rim [28].

The fundamental architecture of SZs in cultured osteoclasts is essentially the same as that of SZs formed on bone, differing from it only in podosome size, number and density [21]. Notably, the overall size and dynamics of SZs depend very much on the chemical and physical properties of the underlying substrate [11], [29]. For example, on bone, SZs are rather small and relatively stable, whereas on vitronectin (VN)-coated glass surfaces, the SZs are highly dynamic and apparently unstable [29]. In addition, on bone, several smaller SZs are found in each cell, while on glass, the corresponding structures fuse into one large peripheral ring, suggesting that osteoclasts can locally degrade bone regions with a subcellular resolution [29]. Considering the apparent confinement of SZs on bone, it appears reasonable to assume that SZ formation and development (and, as a consequence, bone degradation) are tightly regulated by multiple cues or barriers, which are present on the underlying adhesive matrix; hence, variations in the chemical and physical properties of bone can greatly affect degradation rates [30], [31], [32].

To explore the role of osteoclast-matrix adhesion in the regulation of SZ formation and dynamics, we used micro-patterned surfaces, consisting of adhesive, VN-functionalized areas, separated by non-adhesive regions passivated with the co-polymer poly(L-lysine))-*g*-poly(ethylene glycol) (PLL-*g*-PEG). These studies indicated that sealing zone expansion is dependent on the continuity of matrix adhesiveness, and can be arrested by PLL-*g*-PEG-passivated regions as narrow as 900 nm, and demonstrate that fusion of neighbouring SZs cannot occur when the two rings are separated by a non-adhesive barrier.

## Results

### Sealing zone behavior at the interface between VN-functionalized and PLL-g-PEG-passivated surfaces

**Figure 1:**
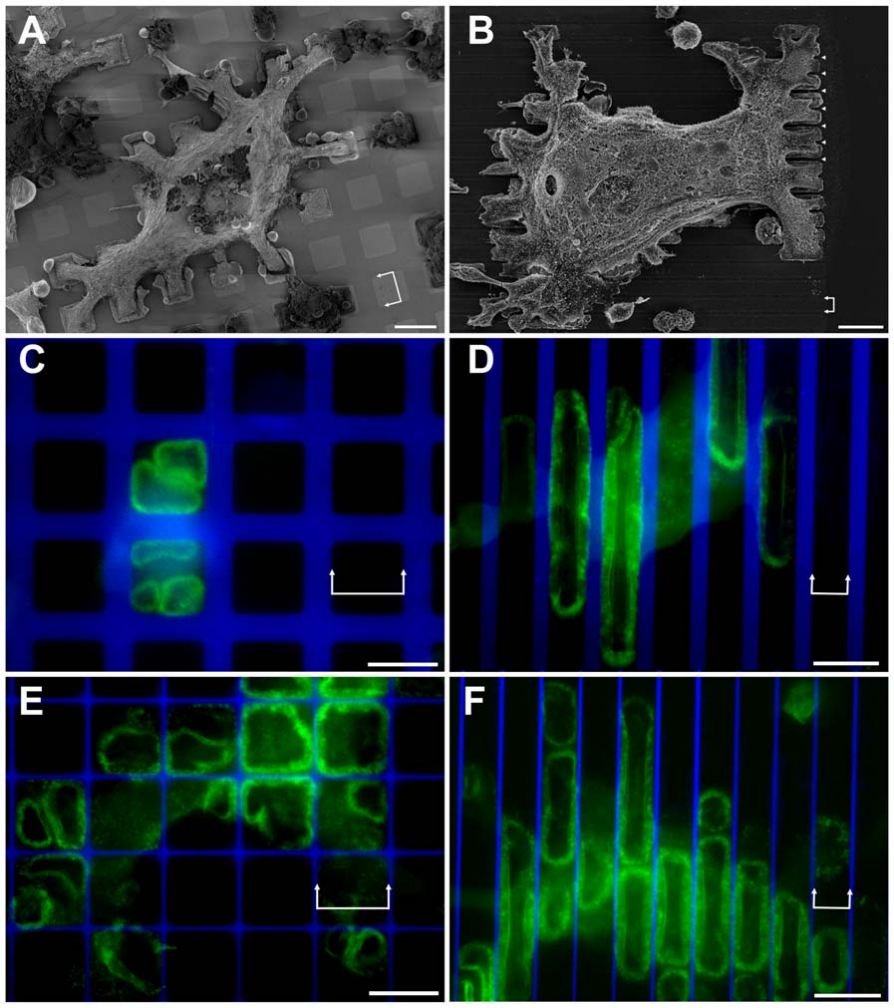
Osteoclasts and sealing zones on vitronectin (VN)/PLL-*g*-PEG micro-patterns. (**a**, **b**) SEM micrographs of differentiated osteoclasts spreading over non-adhesive PLL-*g*-PEG areas on square and striped VN-coated micro-patterns: **a**) 15×15 µm, marked with double arrows, 10 µm-wide barrier; **b**) 6 µm-wide stripes, marked with double arrows, 1.8 µm-wide barrier, marked by arrowheads). The cells were critical point dried after partial removal of the cell body, to enable better detection of the adhesion pattern on the substrate. (**c**, **e**) Osteoclasts growing on 20×20 µm VN patterns separated by (**c**) 8.5 µm- or (**e**) 1 µm-wide PLL-*g*-PEG barriers. Panel **c** shows a single frame from a time- lapse movie of a GFP-actin expressing osteoclast, while in Panels **d-f**, actin was stained with phalloidin-FITC. (**d**, **f**) Osteoclasts growing on 11 µm-wide VN coated stripes separated by (**d**) 4.5 µm- or (**f**) 900 nm-wide PLL-*g*-PEG barriers. Green: GFP-actin/Phalloidin-FITC, Blue: PLL-*g*-PEG-TRITC. Scale bars, 20 µm.

An adaptation of the Molecular Assembly Patterning by Lift-off (MAPL) procedure [7] was used to create micro-patterns of adhesive VN islands with different sizes and shapes, separated by PLL-*g*-PEG coated, non-adhesive areas (Fig. S1). Two different types of adhesive islands were utilized in this study: adhesive islands completely confined by non-adhesive barriers (e.g., adhesive squares of different sizes) and adhesive stripes offering continuous adhesion in one dimension while preventing it in the other. Differentiated Raw 264.7 [21] osteoclasts expressing GFP-actin were plated on such micro-patterned surfaces and monitored by fluorescent time-lapse video microscopy. The cells adhered to the substrate and spread over it, covering multiple adhesive islands, and crossing non-adhesive PLL-*g*-PEG barriers of variable width (Fig. 1a, b). Cell spreading over the surface was somewhat attenuated, yet not blocked by PLL-*g*-PEG barriers of up to 10 µm, results that are comparable to previous reports of fibroblast spreading on similar surfaces [7], [33]. Examination of actin distribution indicated that, in contrast to the whole cell body, SZs were confined to the adhesive VN-coated islands (Fig. 1c, f) and only rarely (<1%, see below) crossed passivated areas.

Examination of osteoclasts spreading on surfaces with passivated barriers varying in width between 0.9–10.0 µm, indicated that arrest of SZ translocation was obtained even with the narrowest PLL-*g*-PEG barrier tested, namely, 0.9 µm.

**Figure 2:**
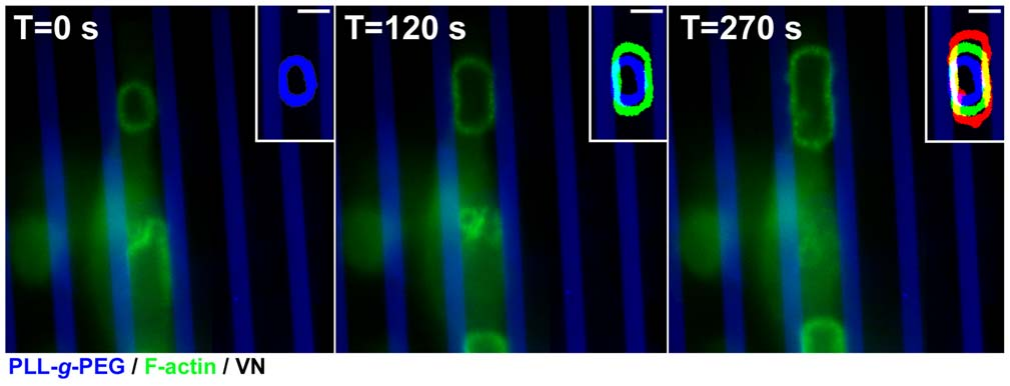
Sealing zone dynamics on micro-patterned surfaces. Time-lapse images of an expanding SZ on a 6 µm-wide adhesive VN-coated stripe flanked by 3 µm-wide PLL-*g*-PEG barriers. Green: GFP-actin; Blue: PLL-*g*-PEG-TRITC. Inserts show overlays of the same ring at three time points, represented by different colours: *t* = 0 s blue; *t* = 120 s green; *t* = 270 s red. Scale bars, 5 µm. Note that as soon as the SZ reaches a passivated area, its outward movement is arrested.

Live cell recording indicated that outward expansion of the SZ rings occurs at varying rates, typically on the order of 1–3 µm/minute (Fig. 2, inserts). However, as soon as the SZs reach the VN/PLL-*g*-PEG interface, expansion is arrested (Fig. 2, and Movie S1). This arrest of SZ expansion was a rather local phenomenon, confined to the regions of the SZ bordering the PLL-*g*-PEG barrier, while the regions of the same SZ, which are associated with the VN-coated area, kept expanding at the normal rate (Fig. 1d, f, and Fig. 2). Furthermore, SZs arrested at the boundary between functionalized and passivated regions proved to be more stable and structurally intact, compared to those formed on uniform VN-coated glass substrates. On the latter surfaces, SZs were apparently unstable and were often fragmented (∼60%, see also [29]), while the percentage of broken SZs at the boundaries between adhesive and non-adhesive regions was <10%.

### Structural properties of sealing zones located at the interface between adhesive and non-adhesive zones

**Figure 3:**
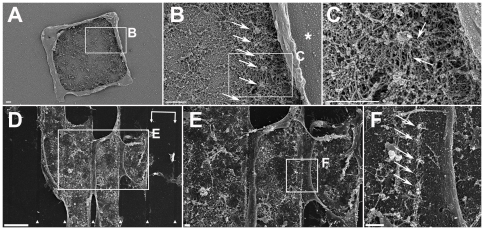
Sealing zone architecture at the VN/PLL-*g*-PEG interface. (**a**–**c**) A ventral membrane (shown at 3 levels of magnification) displaying a sealing zone at the VN/PLL-*g*-PEG interface. (**b**) Arrows point to podosome cores, while the asterisk indicates the border between VN and PLL-*g*-PEG. (**c**) Arrows point to lateral and interconnecting actin fibers anchoring the podosomes to the membrane and connecting them to neighboring podosomes, respectively. (**d**–**f**) A ventral membrane (shown at 3 levels of magnification) displaying a row of podosomes formed at the VN/PLL-*g*-PEG interface. (**d**) 10 µm-wide VN stripes, marked with double arrows; 1 µm-wide barriers, marked by arrowheads. Scale bar, 10 µm. (**e**, **f**) Magnifications of indicated selections. Scale bars, 1 µm. (**f**) Arrows point to podosome cores formed at the VN/PLL-*g*-PEG interface.

To explore at higher resolution the structural consequences of the arrest of SZ expansion at the VN/PLL-*g*-PEG interface, osteoclasts were cultured on the aforementioned micro-patterns, and substrate-attached ventral membranes, obtained after “unroofing” the cells by shear flow, were critical point dried and examined by scanning electron microscopy (SEM) [21], [29]. The boundaries between the passivated and functionalized areas, as well as the different structural elements of the associated SZ, including individual podosomes, their “core bundles”, the actin filaments anchoring them to the membrane (“lateral filaments”), and the actin filaments interconnecting adjacent podosomes could be clearly visualized. In line with previous studies [21], podosomes within SZs displayed a densely-packed actin core bundle (∼300 nm in diameter) surrounded by “lateral” and “interconnecting” filaments (arrows in Fig. 3b and c, respectively). The average core-to-core distance was 442±126 nm (n = 123 podosome pairs from 8 different cells). Interestingly, SZs running along the PLL-*g*-PEG barrier were similar to those formed on uniform, VN-coated surfaces, in terms of podosome architecture, density and inter-spacing ([21], and Fig. 3). In line with the fluorescent microscopy images, the podosomes were strictly confined to the VN-functionalized islands bordering the PLL-*g*-PEG barriers (Fig. 3d, f).

**Figure 4:**
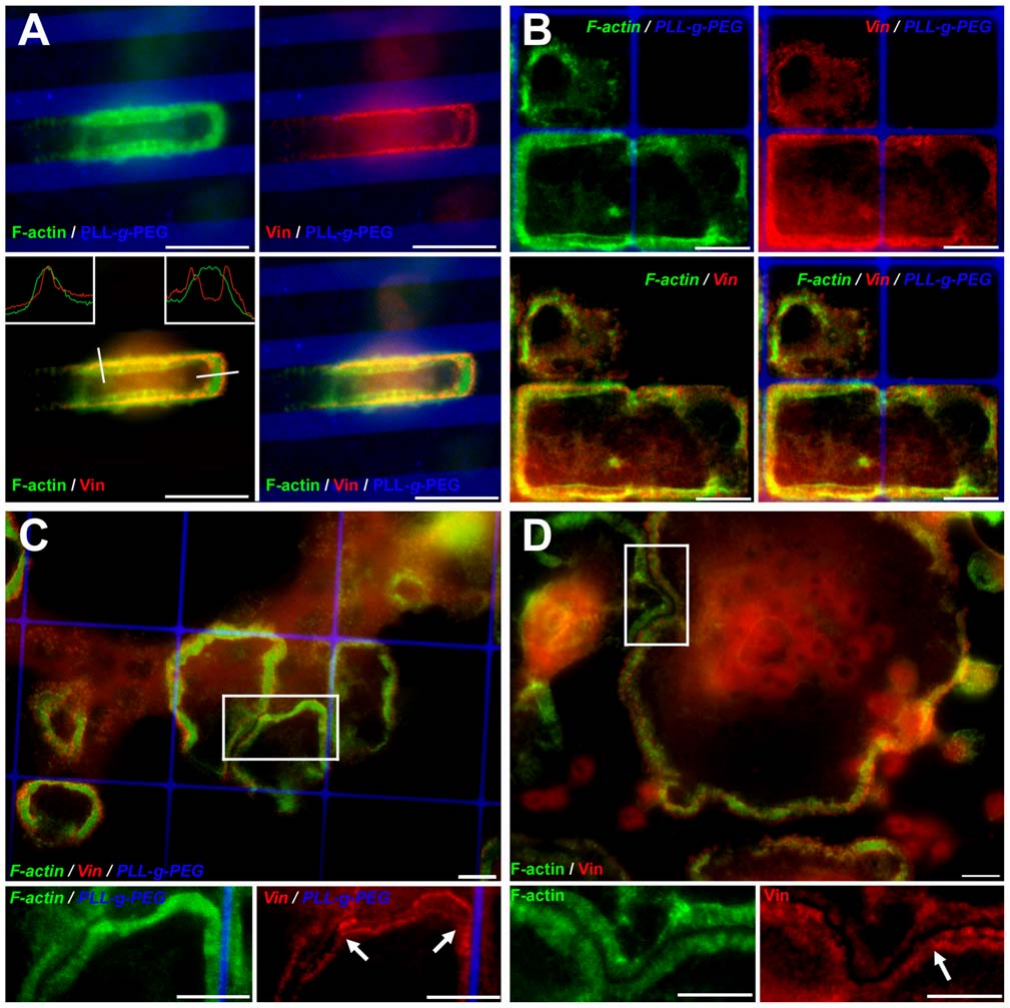
Loss of one vinculin band upon blocking sealing zone expansion. (**a**, **b**) SZ arrested at a VN/PLL-*g*-PEG interface on striped and square micro-patterns: **a**) 5.35 µm-wide VN stripes, separated by 3.3 µm-wide barriers. Inserts: display of line scans of actin and vinculin fluorescence across the SZ at the VN/PLL-*g*-PEG border (left insert) and when on a uniform VN-coated surface (right insert). **b**) 20×20 µm VN squares, separated by 1 µm-wide barriers; (**c**, **d**) SZ arrest by an intercepting SZ within the same cell or a neighbouring cell, respectively. **c**) a SZ on square micro-patterns of 40×40 µm, barrier 1 µm-wide; **d**) a peripheral SZ on non-patterned, VN-coated glass slides. Inserts (bottom) show higher magnification of selected areas, with arrowheads indicating the loss of the vinculin ring in arrested SZ areas, green: phalloidin-FITC; red: anti-vinculin 546 nm; blue: PLL-*g*-PEG-Atto-633. Scale bars, 10 µm.

Examination of the molecular components of the SZ using immunofluorescence microscopy showed changes in the organization of the adhesive vinculin rings. Osteoclasts adhering to a uniform VN surface usually form a SZ consisting of an actin ring, flanked by two adhesive rings an “inner” and an “outer” one [21], [34]. Examination of the SZs arrested at the VN/PLL-*g*-PEG interface, invariably showed only one vinculin ring, located at the very edge of the VN-coated island, without extending into the passivated area (Fig. 4a, b). Intensity measurements indicated that the part of the SZs formed at the boundary contained vinculin levels similar to those present in the outer ring of the same SZs. The intensity of actin, on the other hand, remained the same in the different parts of the SZ. Specifically, the actin intensity ratio between “edge” and “uniform” VN areas was 0.96 ± 0.20 (n =  103 SZs), and the intensity ratio for vinculin (“boundary” vs. the outer vinculin band on uniform VN) was 1.08 ± 0.36 (n = 103 SZs). This suggests that the inner adhesion rings is either lost or fused with the outer ring, accompanied by a major loss of vinculin from these regions of the SZ.

In addition to the apparent loss of vinculin, the spatial relationships between actin and the flanking plaque rings change upon interaction with the PLL-*g*-PEG barrier. Line scans across the SZ in such regions show a “peripheral shift” of the actin ring, partly overlapping the remaining vinculin band, while scans in regions of uniform VN coating confirm a more “central” positioning of actin, between the two vinculin rings (Fig. 4a, inserts). It is noteworthy that the loss of vinculin and the outward shift of the actin ring upon encountering the PLL-*g*-PEG barrier, were observed with different micro-patterns (e.g., squares, Fig 4c), excluding a role for a particular pattern scale or asymmetry in these processes.

Are the changes in SZ structure, induced upon its arrest at the edge on the adhesive area, directly induced by the encounter with the passivated area, or are they secondary to the local arrest of SZ outward translocation? To distinguish between these possibilities, we searched for SZs whose expansion was blocked for reasons other than lack of continuous surface adhesiveness (e.g., contact with a neighbouring SZ, within the same cell, or reaching the cell's edge). Interestingly, we found that the presence of a non-adhesive barrier per se was not the primary inducer of SZ reorganization, and whenever SZ expansion was blocked, vinculin was lost (Fig. 4). In cases where multiple SZs were formed on large adhesive islands (e.g., 40×40 µm squares) only a single adhesive vinculin ring remained upon interaction with the PLL-*g*-PEG barrier, as well as the intercepting SZ (Fig. 4c, arrows in inserts). Similarly, only one residual vinculin ring was observed in cases where the expansion of the SZ is blocked by encountering a neighbouring cell (Fig. 4d, arrow in insert).

**Figure 5:**
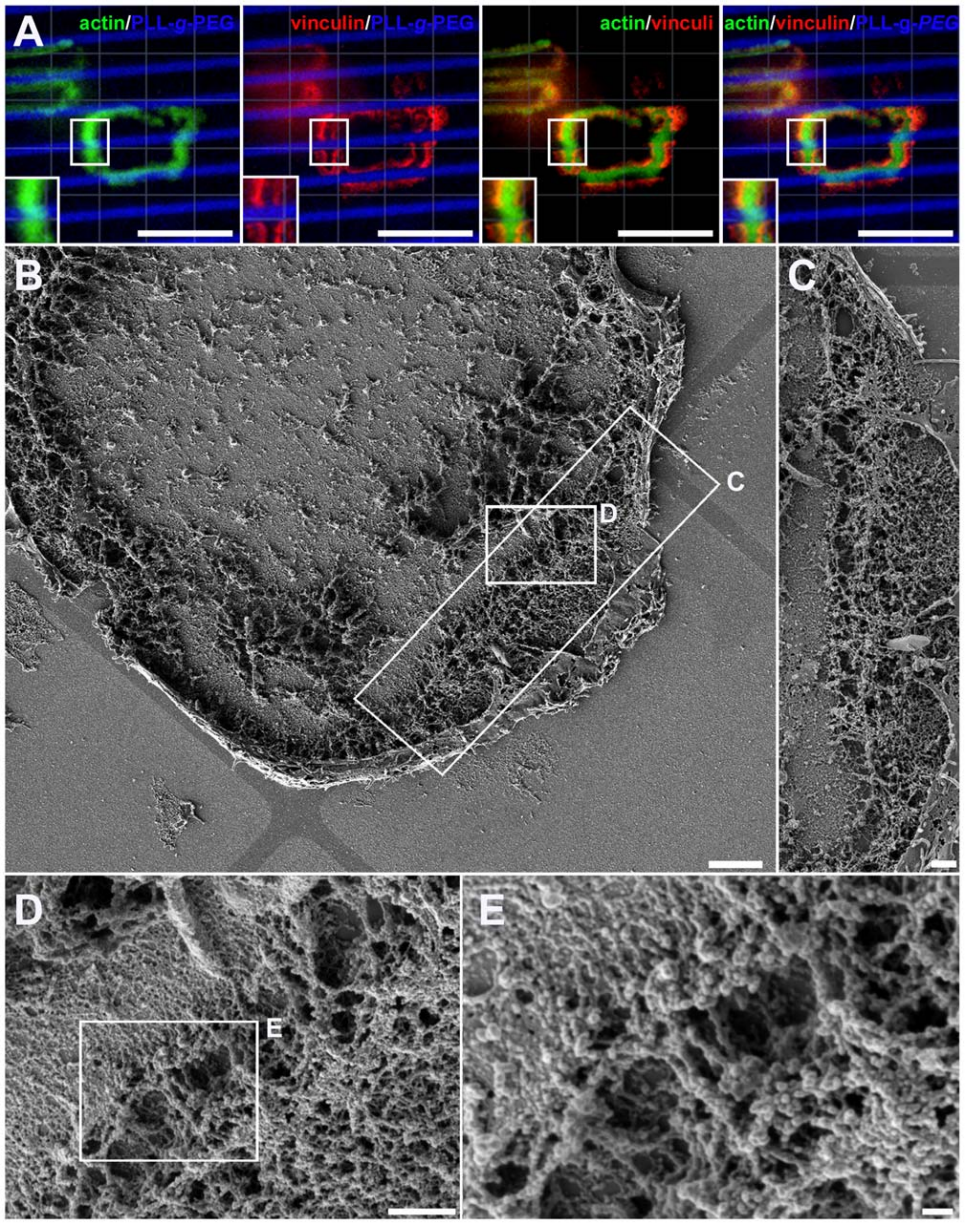
Interconnecting actin fibers bridging over a 1 µm-wide PLL-*g*-PEG barrier. (**a**) SZ formed on striped micro-patterns, bridging a PLL-*g*-PEG barrier (2.8 µm-wide adhesive stripes, barriers 1 µm wide). Note that only the actin component of the SZ bridges the barrier, while the vinculin domains remain restricted to the adhesive areas. Inserts display magnifications of respective selections. Green: Phalloidin-FITC; Red: anti-vinculin 546; Blue: PLL-*g*-PEG-Atto-633. Scale bars, 10 µm. (**b**–**e**) SEM micrograph of a ventral membrane formed on 40×40 µm VN coated micro-patterns displaying a SZ spanning a 1 µm-wide PLL-*g*-PEG barrier by interconnecting actin fibers (shown at 4 levels of magnification). (**c**–**e**) Magnifications of the respective selections in **b** and **d**. Scale bars: (**b**) 3 µm; (**c**, **d**) 1 µm; (**e**) 200 nm. Images in **b**, **d** and **e** were taken after tilting the stage of the SEM by 30°, to facilitate visualization of the area underneath the fibers.

In rare cases (12 out of 1,123 actin rings tested, from movies of 32 cells) the SZ did cross the barrier into the next adhesive field (Fig. 5). These exceptions were observed only when the barrier width was 1.5 µm or less, and time-lapse video microscopy indicated that these events were, indeed, genuine SZ extension events, and were not attributable to the fusion of two SZs, positioned at both side of the narrow barrier, nor to an imperfect passivation. Direct examination of the “bridge areas”, confirmed that the SZ traversing the barrier is enriched with actin filaments, yet is devoid of membrane anchored podosomes (Fig. 5b, e) and vinculin labelling (Fig. 5a). A possible explanation of this phenomenon is that the interconnecting actin filaments might extend from core bundles located at the edge of the passive barrier and “reach out” across the passivated area inducing the formation of a new podosome, through an “inside-out” process (see discussion). This possibility is in line with recent unpublished results of Luxenburg et al. demonstrating that novel podosomes are commonly nucleated of areas enriched with membrane-associated cytoplasmic paxillin.

### Fusion of neighbouring sealing zones depends on the merger of their adhesion domains

**Figure 6:**
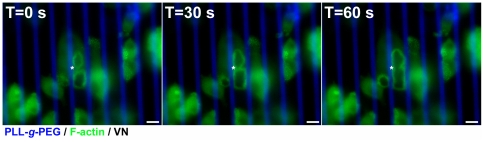
Fusion of sealing zones on VN/PLL-*g*-PEG micro-patterns. Time sequence depicting fusion of two sealing zones on a 10 µm-wide VN stripe separated by a 3.5 µm-wide PLL-*g*-PEG barrier, over 1 min. SZs fuse at the site of contact, followed by a widening of the fusion site toward the edges of the micro-pattern. Green: GFP-actin. Blue: PLL-*g*-PEG-TRITC. Scale bars 10 µm.

SZ fusion, whereby both the actin rings and associated adhesion rings of two SZs merge into one, is a common process in osteoclasts growing on artificial or natural surfaces [11], [28], [29]. However, the specific contributions of the cytoskeletal and adhesion domains of the SZ to this process are not clear. To address this issue, we cultured differentiated osteoclasts on micro-patterned substrates on which adhesive VN stripes (10 µm wide) were separated by 1 µm-wide PLL-*g*-PEG barriers, and compared SZ fusion events across the barriers, with those that take place along the uniform VN-coated stripe. This quantification indicated that fusion events between SZs located on the same VN stripe were highly prominent events (34% of the cases, over a period of 4 h; n = 299 SZ pairs in 17 cells), while not a single fusion event across a barrier of 1 µm, was detected (n = 663 SZ pairs, in 17 cells) over a period of 4 h, see Fig. 6).

Since SZs formed on striped micro-patterns adopt an elongated shape, it was important to exclude the possibility that differences in ring curvature lead to the different behavior. To address this concern, we also monitored fusions of SZs formed on square (10×10 µm^2^) adhesive islands, separated by a 1 µm-wide barrier. Again, we did not observe any SZ fusion across the barrier (n = 187 SZ pairs in 8 cells, over a period of 4 h), indicating that SZ fusion is independent of variations in overall SZ geometry, but strictly dependent on the continuity of matrix adhesion.

## Discussion

Despite the progress made over the last several years in identifying diverse factors regulating the bone-degrading activity of osteoclasts (e.g. [11], [29], [35], [36], [37]), the understanding of the mechanisms whereby the cells sense the external surface at a subcellular resolution and accordingly assemble, a resorptive system, is still poor. In this study, we addressed an essential aspect of this broad topic; namely, the role of matrix adhesiveness in regulating SZ architecture and dynamics. For that purpose, we used synthetic, 2-dimensional micro-patterned surfaces, containing adhesive areas separated by passivated regions. We show here that the continuity of adhesiveness of the underlying surface is of critical importance for SZ formation, extension and fusion, and that non-adhesive barriers as narrow as ∼1 µm can, locally, block these processes. This finding may have major physiological relevance, in view of the extensive physical and chemical heterogeneity of bone surfaces. Thus it was shown that osteoclasts respond differentially to the diverse chemical and physical characteristics of bone, including their molecular composition, the degree of mineralization, rigidity and micro-topography [11], [29], [30], [38], [39]. In view of the key role of SZ stability in bone resorption, we propose that the precise distribution of adhesive and non-adhesive molecules on bone surfaces plays a key role in regulating osteoclasts resorptive function.

Naturally, proper cell adhesion to the matrix is critical for many other tissue scaffolding and signalling processes, such as cell spreading and migration; yet the spatial and temporal resolution of surface sensing occurs at varying scales for different cells and different cellular processes. Thus, cell spreading and migration are dominated by lamellipodial protrusions, which are not strictly adhesion-dependent, and display an intrinsic persistence length in the range of several micrometers [40], [41]. This property enables cells to spread and migrate on heterogeneous surfaces, crossing non-adhesive regions with similar dimensions [1], [13], [33], [42], [43], while SZ expansion and fusion, which are critical for effective bone resorption, are highly adhesion-dependent. Thus, non-adhesive barriers as narrow as ∼1 µm can block SZ growth and fusion. These results are also in line with [39] who showed that surface topography of similar dimensions (few microns) can affect SZ dynamics.

Beyond their significance for understanding the adhesion-mediated regulation of bone metabolism, the results presented here provide novel insights into the basic mechanisms regulating SZ formation and dynamics. As shown in this and in previous studies [28], [34], SZ growth occurs via two primary processes: ring expansion and ring fusion. Both processes lead to the formation of large SZs, running along the cell periphery [28]. Ring expansion occurs through a “treadmill-like process”, whereby new podosomes are formed at the ring's periphery and, concomitantly, “inner” podosomes are lost. As demonstrated in this paper, the process of new podosome formation appears to be strictly adhesion-dependent (integrin-mediated), so that when the outer adhesion ring of a SZ reaches the edge of an adhesive area, the outward translocation is essentially halted. It is noteworthy that the width of the non-adhesive area that can block ring extension (∼1 µm), roughly matches the “footprint” diameter of a single row of podosomes, including the surrounding actin “cloud” [21].

The present study also suggests that beyond the local regulation of SZ expansion, this process can also be regulated at longer-range levels, manifested here by the rare, but highly significant capability of SZ to bypass the inhibitory action of narrow barriers (Fig. 5), suggesting that the interconnecting actin fibers can extend beyond the confines of the podosome contours [21], enabling them to reach close to the ventral membrane, beyond the passivated region and link to podosomes located at these regions, practically offering an inside-out linkage.

An additional observation, made here, is related to the relationships and interplay between the cytoskeletal domain of the SZ (including the podosome cores and associated actin filaments), and the adhesion domain (namely, integrin complexed with the different plaque proteins). Specifically, the loss of one vinculin-rich ring, upon arrest of SZ expansion, discussed above, shows that although the adhesion complexes are essential for podosome formation and SZ extension, arrest of outward translocation of the cytoskeletal structures can affect the assembly and stability of the adhesion complexes, irrespective of the reason for this arrest. The mechanism underlying this phenomenon is not clear, and we did not directly address it in this study, yet we would like to discuss here the possibility that SZ translocation and arrest, as well as modulation of the adhesion plaque, are both driven by mechanical force, and regulated by it.

The mechanosensitivity of integrin adhesions, in a variety of cell types, has attracted much attention in recent years [44], [45], [46], [47], [48], [49], [50], [51], [52] Studies of focal adhesions and the associated contractile actomyosin stress fibers, indicated that the integrity of the adhesion sites depends on the contractile forces applied to them, either by the endogenous cytoskeleton contractility [53], [54], or by external mechanical perturbations [55]. Thus, relaxation of actomyosin contraction, using myosin II inhibitors or Rho kinase inhibitors, leads to deterioration of focal adhesions and the associated stress fibers [56], [57]. Evidently, the cytoskeletal architecture of the SZ with the associated podosomes, is very different from that of the stress fiber-focal adhesion system of fibroblasts and epithelial cells, yet the effect of perturbations such as those addressed in this study, suggest the possibility that mechanical forces play an important role in regulating SZ structure and dynamics.

**Figure 7:**
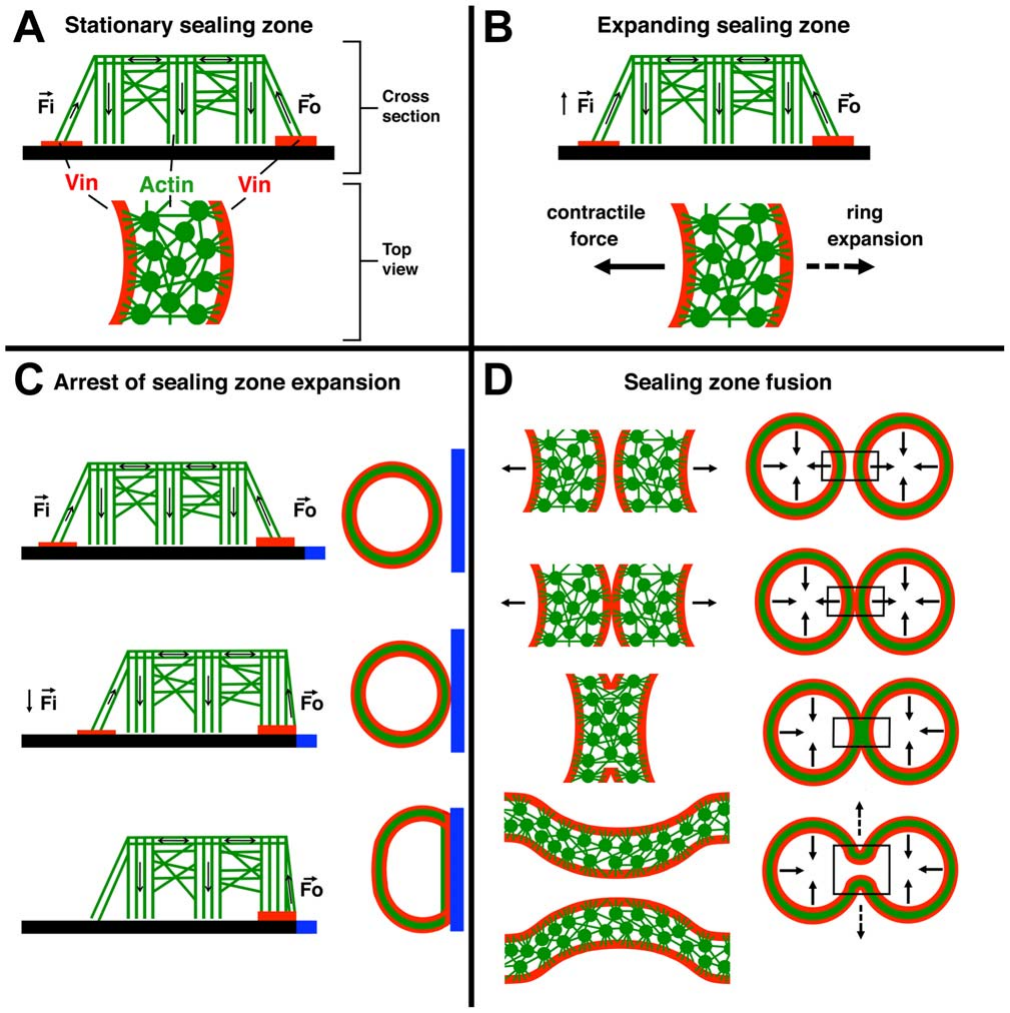
Schematic drawing depicting a possible model for force associated regulation of SZ formation, expansion and fusion. Podosomes are displayed in cross-section, with vertical stripes representing the branched actin core bundle, and horizontal lines indicating the interconnecting actin fibers; top view shows podosome circular actin cores and centrifugally expanding actin fibers either anchoring the cores to the membrane, or interconnecting them. Adhesive plaque areas are represented in red, while black bars represent the adhesive surface. Arrows display forces (Fi  =  force acting on the inner plaque band and Fo  =  force acting on the outer plaque band). (**a**) Cross-section and top view of a SZ. (**b**) A possible two-force regime balancing SZ expansion and integrity, responsible for the force-dependent formation of the inner and outer adhesive plaque domains. (**c**) Model indicating the force-dependent loss of the inner plaque band upon arrest of SZ expansion. The blue bar represents the PLL*-g-*PEG barrier. (**d**) Force- dependent model of SZ fusion, where fusion of the two outer plaque bands is followed by the interlinking of the podosome cores of the two rings, leading to local cancellation of the net force. This, in turn, finally leads to the local disassembly of the podosomes, and the fusion of the two rings.

Figure 7, inspired by the current data, illustrates a possible model for the role of mechanical forces in SZ formation and reorganization. We propose that SZ mechanics are driven by three main processes: contraction of the actin-rich ring of the SZ, mechanical forces generated by actin polymerization in the podosome cores pulling on the lateral fibers, which interconnect them to the adhesion plaques, and “dragging forces” generated by the outward translocation of the SZs. These forces, working in concert, may regulate the maintenance and stability of the adhesion ring, and the expansion of the SZ.

The mechanisms underlying these three force-generating processes, and their possible effect on SZ dynamics deserve a brief discussion here. The mechanical properties of the cytoskeletal ring of the SZ have not been thoroughly investigated; yet it has been demonstrated that myosin II is associated with this structure [58], and addition of actomyosin inhibitors to cultured osteoclasts was found to destabilize the SZ, without disrupting individual podosomes (Fig. S2). Naturally, such “centripetal” forces, applied to the array of podosome cores at the SZ, might predominantly increase stress at the “outer” adhesion ring. Less obvious, yet potentially important, are forces produced due to actin polymerization within the SZ cytoskeletal domain. It has been shown that podosome cores are dynamic structures undergoing constant, “inward” treadmilling [28]. This process may generate “pulling forces” that are mediated by the lateral actin filaments to the adhesion rings of the SZ. Finally, the outward translocation of the SZ ring may also produce a centripetal “dragging force”, applied to the plasma membrane of the inner ring, via the lateral fibers.

How can these mechanical forces affect SZ dynamics? We would like to consider here the possibility that increased tension at the cytoskeleton-membrane interface in the SZ provides a local assembly cue, while reduction in force, leads to local disassembly of the adhesion ring, similar to the effect of such mechanical perturbations on focal adhesions [55]. Such mechanisms might explain the preferential outward translocation of the SZ (mainly driven by the higher tensions applied to the outer adhesion ring), and the dependence of the inner adhesion ring on SZ translocation (which preferentially affects the inner adhesion ring (Fig. 4 and Fig. 7c).

The suggested force-dependent mechanism is also consistent with our finding that not only the formation and expansion of SZs, but also their fusion, strictly depend on continuous surface adhesiveness. The SZ fusion process occurs when two expanding SZs touch each other, and their adhesion and cytoskeletal domains locally fuse [11], [28]. As illustrated in Figure 7 d, a local interaction between the two rings may lead to local arrest in their expansion, thereby inducing disassembly of their inner adhesion rings. The contractile forces, pulling in opposite directions, will then drastically reduce the forces transmitted to adhesion domains at the contact point, leading to their local disassembly and, consequently, to SZ fusion (Fig. 7 d). By culturing osteoclasts on VN/PLL-*g*-PEG micro-patterns (PLL-*g*-PEG spacing of 1 µm), we show that merger of the SZs at the level of the outer adhesion ring is required (Fig. 6).

These considerations are consistent with the data shown here and elsewhere [28], [55], [58], although confirmation of the validity of the force-driven mechanism depicted in Figure 7 will require direct experimental substantiation. In any case, the effects of surface adhesion on SZ dynamics and architecture, described herein, add fundamental information on osteoclast and SZ biology, and may thereby deepen our understanding of the mechanisms regulating bone remodelling.

## Materials and Methods

### Substrate patterning

Micro-patterned glass substrates were prepared, using an adaptation of the previously published Molecular Assembly Patterning by Lift-off (MAPL) technique [7]. No. 1 glass cover slips with dimensions of 22×40 mm (Warner Instruments, Harvard Apparatus GmbH) were sputter-coated with a transparent layer of ∼10 nm niobium oxide (Nb_2_O_5_) using a Leybold direct-current magnetron Z600 sputtering unit (PSI). After removing adsorbed H_2_O by drying the substrate on a hot plate (180°C, 5 min), S1818 positive photoresist (Shipley) was spin-coated on the substrate at 3500 rpm for 90 s, leading to a resist coating of ∼ 2 µm thickness before the samples were post-backed at 100°C for 2 min. The photoresist was illuminated through a chromium mask (Delta Mask), using a Karl Suss MA6 mask aligner as a UV source. A dose of 135 mJ/cm2 was applied prior to development of the substrates using MF-319 developer; samples were exposed for 1 min. The patterned glass cover slips were rinsed with ddH_2_O, and diced into 1×1 cm chips using a diamond cutter.

After photolithography, the pre-patterned substrates carrying a micro-pattern of S1818 photoresist on an Nb_2_O_5_ background were subjected to molecular assembly pattern by lift-off. Prior to the adsorption of the non-fouling Poly(L-lysine)-graft-poly(ethylene glycol) polymer (PLL*-g-*PEG, SuSoS AG) the samples were plasma-cleaned for 15 s using air plasma (10^−5^ bar, PDC-32G-2, Harrick Plasma). The sample was immersed in an aqueous solution of TRITC- or ATTO 633-labeled PLL*-g-*PEG copolymer (1 mg/ml in 1×PBS, 60 min) immediately after plasma cleaning. Subsequently, the samples were rinsed with ddH_2_O and a lift-off experiment was performed, in order to expose the Nb_2_O_5_ underlying the photoresist pre-pattern. The samples were exposed to a series of different dilutions of 1-Methyl-2-pyrrolidone (NMP for peptide synthesis, Fluka-Chemie AG): 20 s in a 1:2 dilution of NMP and ultrapure H_2_O in an ultrasonic bath; 10 s in ultrapure H_2_O, 1 min in NMP in an ultrasonic bath; 5 min in fresh NMP in an ultrasonic bath; and 1 min in a 1∶1 dilution of NMP and ultrapure water in an ultrasonic bath. Finally, the samples were rinsed for 5 min in an ultrapure water bath, and agitated with a magnetic stirrer, leading to a PLL*-g-*PEG patterned, niobium oxide-coated glass slide. Finally, the patterns were backfilled with a 10 µg/ml aqueous solution of vitronectin for 20 min.

### Tissue culture and immunohistochemistry

RAW 264.7 cells stably expressing GFP-actin [21] were cultured in tissue culture flasks (60 ml; TPP) and differentiated in 24-well test plates (TPP). To induce differentiation, cells were cultured in alpha MEM with Earle's salts, L-glutamine and NaHCO3 supplemented with fetal bovine serum (FBS, 10%; Gibco), antibiotics (Biological Industries), recombinant soluble receptor activator of NFκB ligand (RANK-L, 10 ng/mL; R&D), and macrophage colony-stimulating factor (M-CSF, 10 ng/ml; R&D), at 37°C in a 5% CO_2_ humidified atmosphere for three days. Once differentiated, the cells were removed with EDTA (10 mM) for 10 min, and then plated for 36 h on either micro-patterned or plasma-treated and VN- coated glass slides.

After o/n cultivation on the respective substrate, cells were fixed for 3 min in warm 3% PFA (Merck) with 0.5% Triton X-100 (Fluka-Chemie AG) followed by 3% PFA alone for an additional 40 min. After fixation, cells were washed 3 times with PBS (pH 7.4) and incubated with primary antibody for 40 min, washed 3 times in PBS, and incubated for an additional 40 min with the secondary antibody. Finally, samples were washed 3 times in PBS and mounted using IMMU-MOUNT (Thermo Shandon).

Primary antibody: monoclonal anti-human vinculin (hVIN-1) antibody produced in mouse (Sigma). Secondary antibody: goat anti-mouse secondary antibody labelled either with Alexa 488 or Alexa 546 fluorescent tags (Invitrogen - Molecular Probes). Actin was labelled with phalloidin, and conjugated to FITC (Sigma).

For live-cell imaging, differentiated RAW 264.7 osteoclasts replated onto vitronectin-coated or micro-patterned glass substrates, as described above, were probed for up to 12 h, starting 6 h or 18 h hours after replating. Data was acquired at 30 s intervals at 37°C in a 5% CO_2_ humidified atmosphere_,_ using a Zeiss Live Cell Station microscope (Carl Zeiss AG) equipped with a 63x 1.4NA Oil DICIII Plan Apochromat objective.

### Ventral membrane preparation for SEM microscopy

To characterize the adhesive structures in a cellular environment, we developed a sample preparation technique involving high-resolution, three-dimensional electron microscopy. The procedure is an adaptation of published procedures [59], [60], entailing the unroofing of the cell basal portion, while preserving the components' three-dimensional organization and immunogenicity. In brief, osteoclasts were exposed to H-PHEM solution for 60 s prior to unroofing by shear stress. Exposed ventral membranes were immediately fixed with warm glutaraldehyde (2%; Electron Microscopy Sciences – EMS) in PBS for 30 min. Cells were then washed three times for 5 min in PBS and sodium cacodylate buffer (0.1 M; Merck) containing CaCl_2_ (5 mM: pH 7.3), followed by post-fixation with OsO4 (1%, EMS) in cacodylate buffer for 60 min. After fixation, samples were washed (3×5 min) with cacodylate buffer and H_2_O, respectively. The preparations were then incubated with tannic acid (1%; Merck) in H_2_O for 5 min, and washed in H_2_O (3×5 min). Dehydration in increasing concentrations of reagent-grade EtOH (2×5 min in 50% and 70%, and 2×10 min in 95%, and 100%) was followed by drying with a critical point dryer (CPD30; Bal-Tec AG). Samples were coated with 4–6 nm Cr using a sputter coater (K575X; Emitech), and visualized in the high-resolution SEM Ultra 55 and the Gemini 1530 FEG microscopes (Carl Zeiss AG).

## Supporting Information

Figure S1 **Schematic drawing, depicting the MAPL process (adapted for protein micro-patterning).** 1) Niobia coated glass slides (gray bars) are patterned with photoresist (red bars) by applying UV light (purple arrows) through a photomask (black cubes). 2) This is followed by a development step dissolving the exposed photoresist and leading to micro-patterns of the remaining photoresist. 3) The patterned chips are immersed in a PLL-*g*-PEG solution (vertical black lines) followed by a lift-off step in organic solvents releasing the remaining photoresist leading to PLL-*g*-PEG micro-patterns on an empty Nb_2_O_5_ background (4). 5) This new formed background is then backfilled using a 10 µg/ml VN solution (green spirals) leading to the final VN/PLL-*g*-PEG micro-patterns that are then used in culture experiments (6).
(TIF)

Figure S2 **Sealing zone disassembly into individual podosomes as a consequence of the loss of myosin contractility after blebbistatin treatment.** Green: Actin-GFP, Blue: PLL-*g*-PEG-TRITC, scale bars represent 10 µm each. Time series showing the gradual disassembly of two SZs formed on 20×20 µm2 adhesive VN areas separated by 5 µm wide PLL-*g*-PEG barriers, into individual podosomes after treatment with 50 µM of blebbistatin. T indicates the time in minutes after blebbistatin treatment, arrows indicate individual podosomes.
(TIF)

Movie S1 **SZ dynamics on striped VN/PLL-** ***g*** **-PEG micro-patterns.** Formation, expansion, fusion and brake downs of multiple SZs formed on 9.5 µm wide VN stripes separated by 2.5 µm PLL-*g*-PEG barriers over 50 min. Time resolution: 10 s, Green: GFP-actin; Blue: PLL-*g*-PEG-TRITC. Note the continuous expansion of SZs along the VN stripes while their outward movement is immediately arrested upon interaction with the PLL-*g*-PEG. Also note that SZ fusion only occurs between neighbouring SZs on the same VN stripe.
(AVI)
