# Supplementary figures and images for: Substrate Adhesion Regulates Sealing Zone Architecture and Dynamics in Cultured Osteoclasts
Source: PLoS One. 2011 Dec 5;6(12):e28583. doi: 10.1371/journal.pone.0028583 (PMC3230604; doi:10.1371/journal.pone.0028583)

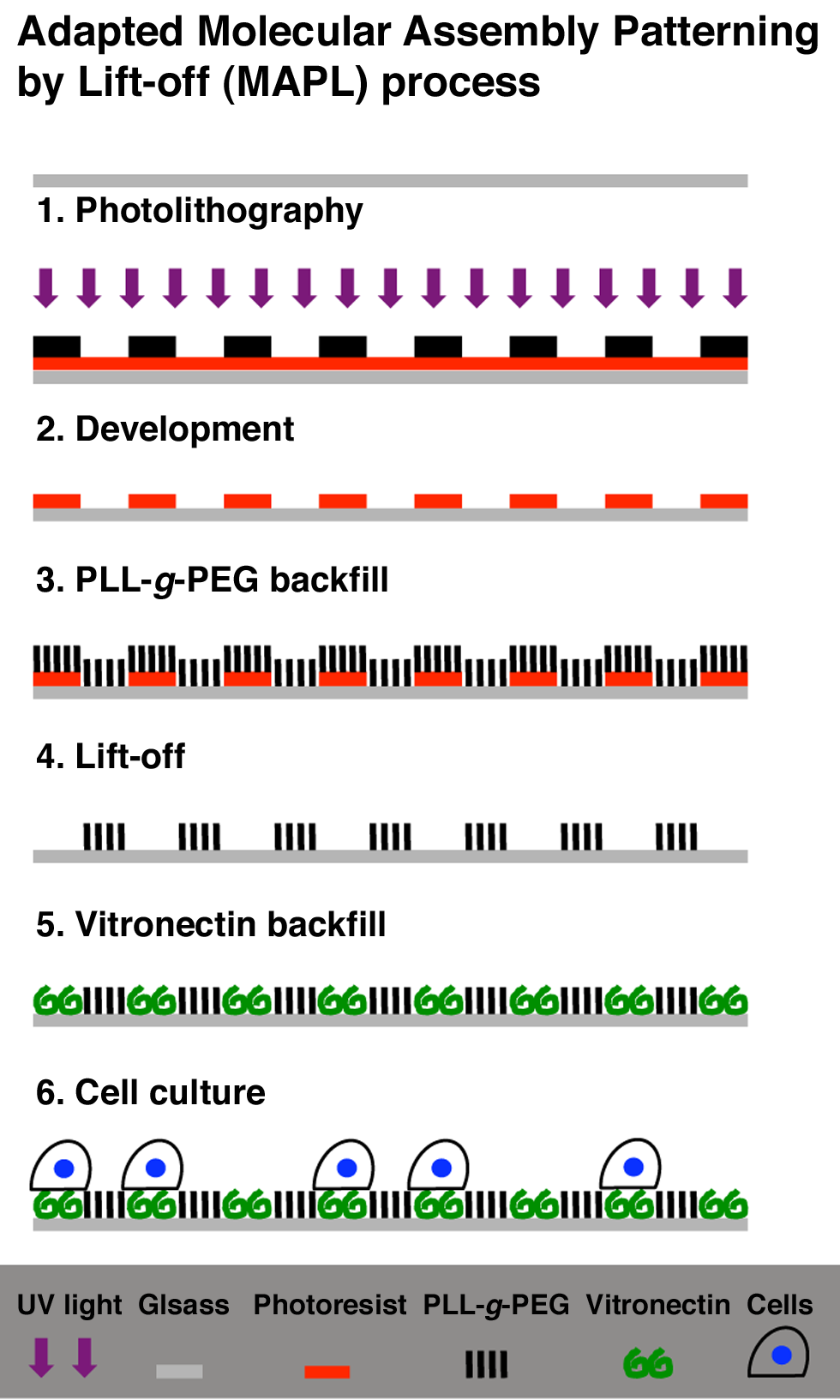

Supplement: Figure S1 — Schematic drawing, depicting the MAPL process (adapted for protein micro-patterning). 1) Niobia coated glass slides (gray bars) are patterned with photoresist (red bars) by applying UV light (purple arrows) through a photomask (black cubes). 2) This is followed by a development step dissolving the exposed photoresist and leading to micro-patterns of the remaining photoresist. 3) The patterned chips are immersed in a PLL-g-PEG solution (vertical black lines) followed by a lift-off step in organic solvents releasing the remaining photoresist leading to PLL-g-PEG micro-patterns on an empty Nb2O5 background (4). 5) This new formed background is then backfilled using a 10 µg/ml VN solution (green spirals) leading to the final VN/PLL-g-PEG micro-patterns that are then used in culture experiments (6). (TIF) [file pone.0028583.s001.tif]

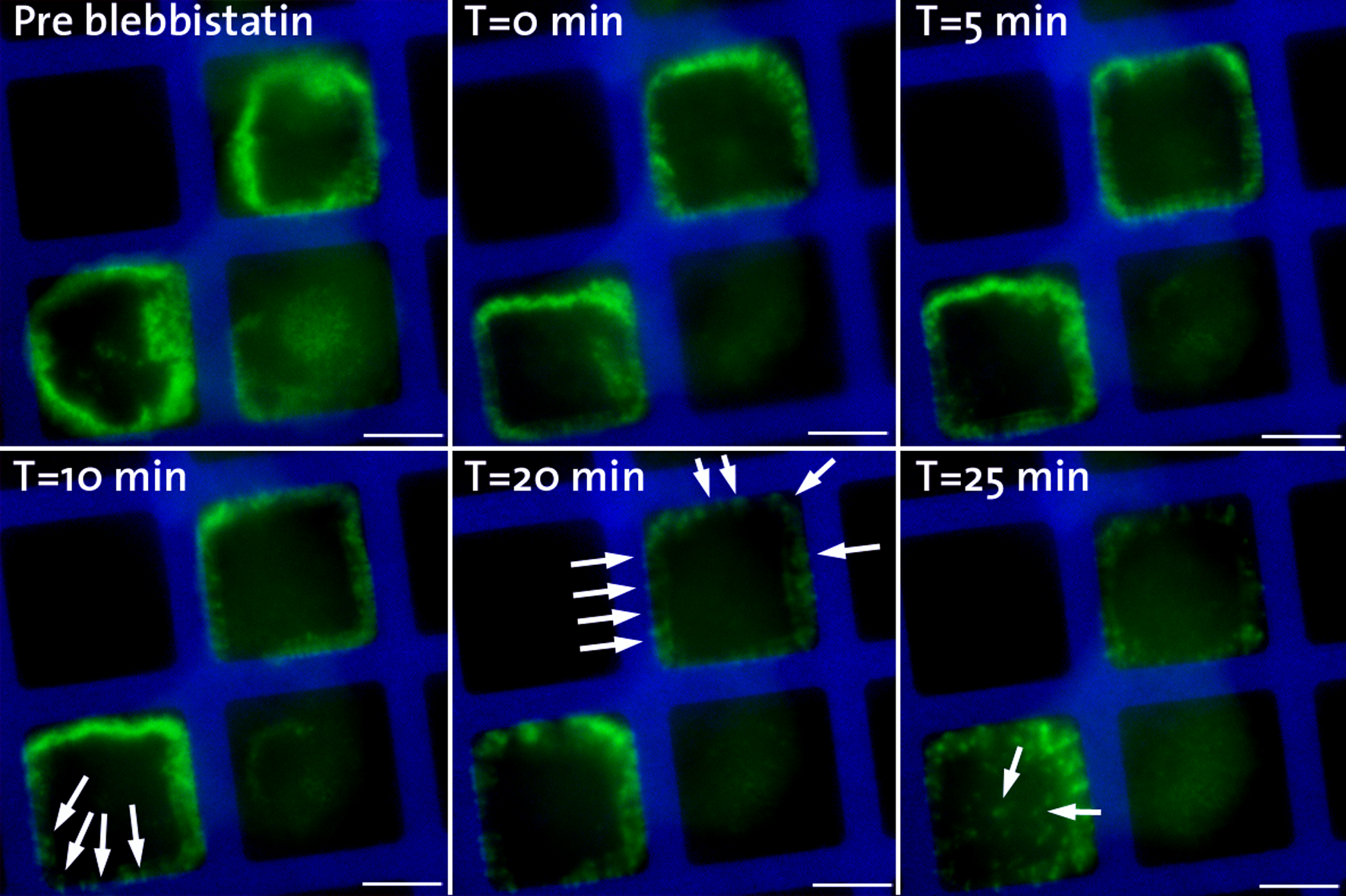

Supplement: Figure S2 — Sealing zone disassembly into individual podosomes as a consequence of the loss of myosin contractility after blebbistatin treatment. Green: Actin-GFP, Blue: PLL-g-PEG-TRITC, scale bars represent 10 µm each. Time series showing the gradual disassembly of two SZs formed on 20×20 µm2 adhesive VN areas separated by 5 µm wide PLL-g-PEG barriers, into individual podosomes after treatment with 50 µM of blebbistatin. T indicates the time in minutes after blebbistatin treatment, arrows indicate individual podosomes. (TIF) [file pone.0028583.s002.tif]
